# Applications of Aptamer-Bound Nanomaterials in Cancer Therapy

**DOI:** 10.3390/bios11090344

**Published:** 2021-09-18

**Authors:** Liangxi Zhu, Jingzhou Zhao, Zhukang Guo, Yuan Liu, Hui Chen, Zhu Chen, Nongyue He

**Affiliations:** 1 State Key Laboratory of Bioelectronics, School of Biological Science and Medical Engineering, Southeast University, Nanjing 210096, China; zhuliangxi@seu.edu.cn (L.Z.); zhaojingzhou@seu.edu.cn (J.Z.); guo724kk@foxmail.com (Z.G.); yuanliu11@seu.edu.cn (Y.L.); 2 Hunan Key Laboratory of Biomedical Nanomaterials and Devices, Hunan University of Technology, Zhuzhou 412007, China; huier_88@vip.163.com (H.C.); chenzhu@hut.edu.cn (Z.C.)

**Keywords:** aptamers, nanomaterials, cancer, treatment, targeting

## Abstract

Cancer is still a major disease that threatens human life. Although traditional cancer treatment methods are widely used, they still have many disadvantages. Aptamers, owing to their small size, low toxicity, good specificity, and excellent biocompatibility, have been widely applied in biomedical areas. Therefore, the combination of nanomaterials with aptamers offers a new method for cancer treatment. First, we briefly introduce the situation of cancer treatment and aptamers. Then, we discuss the application of aptamers in breast cancer treatment, lung cancer treatment, and other cancer treatment methods. Finally, perspectives on challenges and future applications of aptamers in cancer therapy are discussed.

## 1. Introduction

Cancer is one of the key threats to human health. Cancer is a disease caused by abnormal cell growth. Cancer cells may spread to different tissues and organs [1]. According to statistics from the World Health Organization, tens of millions of people worldwide are diagnosed with cancer each year. Most cancers are caused by the external environment, such as smoking, radiation, and environmental pollution. Until now, the main way to treat cancer in developed countries has been chemotherapy, radiotherapy, and surgical treatment [2,3]. However, these cancer treatments have a series of problems, such as easy recurrence, poor treatment effects, and large side effects. Hence, people are looking forward to developing new strategies to treat cancers.

In recent years, nanotechnology has developed rapidly, and more and more nanomaterials with excellent physical and chemical properties have been discovered [4] in the fields of electronics, magnetics, and optics. Many nanomaterials have been used by researchers in the field of biomedicine [5,6,7,8,9,10,11,12,13]. For example, nanomaterials with photothermal conversion properties are used for tumor treatment [14,15,16] and mesoporous materials are used for drug delivery [17,18,19], while magnetic nanomaterials have been found to have excellent superparamagnetic properties, and carbon nanomaterials have been found to feature broad absorbance regions [20,21,22,23]. The focus on nanomaterial applications has been moving from the cellular level toward the tissue level, including cellular imaging, drug delivery, and cancer diagnosis/therapy. Via the enhanced permeability and retention (EPR) effect, most nanomaterials accumulate in a targeted region. People have also proposed various strategies to improve the flow of nanomaterials in blood and improve their stability and biocompatibility [24,25]. However, the EPR effect is not always effective, and the targeting ability of nanomaterials is not good. These problems are accompanied by the issue of harmful toxicity to nontarget tissues or organs. Thus, this problem hinders the development of nanomaterials in bioapplications. Therefore, active targeting strategies have been considered as more powerful tools for cancer treatments, and much attention has been focused on selecting targeted moieties to endow antitumor agents with specific recognition of tumor cells via the affinity between ligands and receptors. There are many receptors overexpressed on the membrane of tumor cells. Based on this, various specific ligands have been discovered, including antibodies, transferrin, folic acid, and peptides. Apart from these ligands, aptamers have become one kind of the most attractive biomolecules.

**Figure 1:**
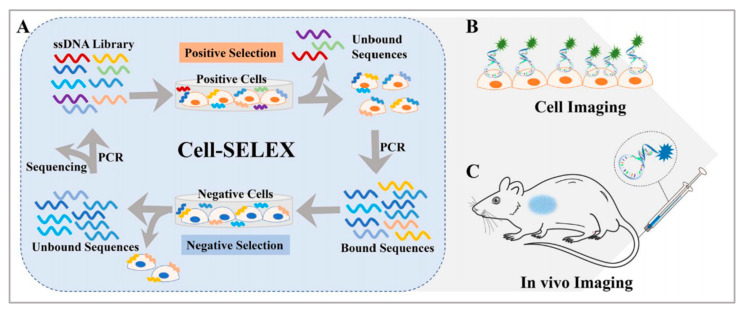
(**A**) Basic principle of the cell-SELEX method and applications for (**B**) cell imaging and (**C**) in vivo imaging. Reprinted with permission from [29]. Copyright © 2020 American Chemical Society.

It is well known that aptamers have a highly selective recognition ability that helps them recognize targets [26,27,28]. Aptamers are single-stranded DNAs or RNAs evolved through systematic evolution of ligands by exponential enrichment (SELEX) technology [29,30] and they are widely employed, as shown in Figure 1. Aptamers can fold into various secondary structures, further forming three-dimensional structures, which contribute to the interaction between aptamers and their targets through various forces, such as hydrophobic interaction and electrostatic attraction [31,32]. Moreover, compared with traditional targeting ligands, aptamers have their advantages, such as good reproducibility, convenient modification, small size, low toxicity, good stability, and low molecular weight [33,34,35]. In addition, aptamers have higher rates of tumor penetration, retention, and homogenous distribution, while the attachment process of aptamers to the surface of nanomaterials is more amenable and reproducible. In principle, an aptamer sequence can be deleted, added, or united flexibly to endow aptamers with tunable recognition ability to better identify targets. Stimulus–response strategies including light, pH, ligand binding, and other cues which have been developed into the design of aptamer-based nanomaterials [27,28,29]. In short, these merits make aptamers ideal candidates for disease diagnosis and therapeutics owing to their ability to deliver therapeutic cargoes into cancer cells, diseased tissue, and organs. Hence, the introduction of aptamers into the construction of nanomaterial systems will promote their bioapplications.

**Scheme 1:**
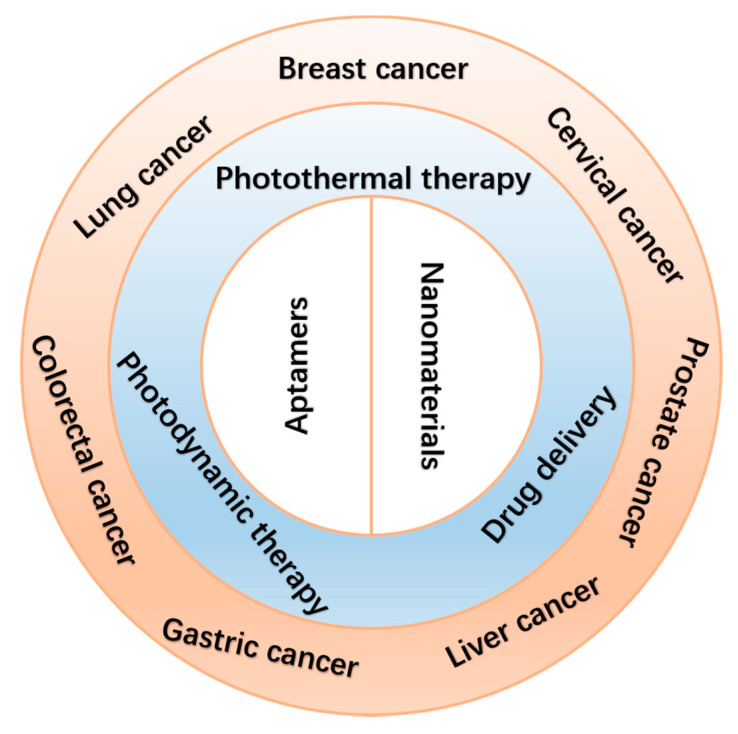
Schematic illustration of the applications of aptamer-bound nanomaterials in cancer therapy.

In this review, we summarize several current applications of aptamer-bound nanomaterials in cancer therapy (Scheme 1). Today, there are various methods to treat cancer. Here, however, we mainly focus on photothermal therapy (PTT), photodynamic therapy (PDT), and improved drug delivery systems (DDSs). We chose several common cancers, including breast cancer, lung cancer, liver cancer, cervical cancer, gastric cancer, colorectal cancer, and prostate cancer. In the first section, we introduce the combination of aptamers with nanomaterials in different cancer therapies. Next, we list several commonly used nanomaterials in cancer therapies (Table 1). Finally, we discuss perspectives on challenges and future applications of aptamer-bound nanomaterials in cancer therapy.

## 2. Therapeutic Method

### 2.1. Photothermal Thearpy

PTT has gained much attention in the field of cancer therapy since Goldman used laser irradiation to remove tumors in 1966 [36,37,38,39,40,41,42,43]. Owing to its minimal toxicity, noninvasiveness, convenient operation, and low recurrence, PTT showed great promise for cancer therapy. After photosensitizing agents accumulate in the tumor site, a near-infrared (NIR) laser is used. Because of the high photothermal conversion efficiency of photosensitizing agents, the absorbed optical energy is converted into thermal energy, leading to either partial or complete ablation of the target tissues. For effective ablation of the tumors, PTT often requires the tumor center to reach high temperatures (≥50 ℃). Because of the deep-tissue penetration of NIR light, various organic and inorganic phototherapeutic agents have been developed. For example, Guo et al. prepared B quantum dots (BQDs) with good biocompatibility. BQDs can effectively produce photothermal effects under NIR light irradiation. Both in vitro and in vivo experimental studies showed that BQDs-PEG can significantly kill cancer cells and inhibit tumor growth through photothermal effects [36]. Leng et al. synthesized core-shell and dumbbell-like gold nanorods-Cu_7_S_4_ heterostructures. Both gold nanorods-Cu_7_S_4_ heterostructures exhibited significantly enhanced photothermal conversion efficiency (η = 56% and 62%) and good photothermal stability. The in vitro photothermal ablation of cancer cells featured low cytotoxicity and effective photothermal treatments [37]. Wang et al. developed a new dual-targeted small-molecule organic photothermal agent for enhanced photothermal therapy, which can target both biotin and mitochondria. The in vivo photothermal therapy experiments indicated that the dual-targeted photothermal agent performed much better in tumor inhibition [39].

### 2.2. Photodynamic Therapy

During the past few decades, PDT has been an attractive therapeutic method for tumor therapy [44,45,46,47,48] because it is noninvasive, spatially selective, and has negligible toxicity. After photosensitizers accumulate in the tumor site, reactive oxygen species (ROS) are produced by NIR laser irradiation. Toxic ROS damages tumor cells via the oxidation of protein, DNA, or RNA. Different photosensitizers have been prepared to overcome both accumulation issues and the hypoxic environment in the tumor. For example, Zhou et al. developed an activatable ROS generation system by modulating a biochemical reaction between linoleic acid hydroperoxide and catalytic iron (II) ions. The engineered nanoparticles were capable of inducing apoptotic cancer death both in vitro and in vivo through ROS generation [46]. Yang et al. prepared a Pt nanoenzyme with functionalized nanoplatform black phosphorus/Pt-Ce6/PEG nanosheets for synergistic photothermal and enhanced photodynamic therapy, in which the Pt nanoenzyme would decompose H_2_O_2_ into oxygen to enhance the photodynamic effect [48].

### 2.3. Drug Delivery Systems

A DDS can be defined as a method or process using the principles of chemistry, engineering, and biology for administering pharmaceutical compounds. Due to their special properties, DDSs have shown tremendous promise to improve the diagnostic and therapeutic effects of drugs, especially in enhancing the pharmaceutical effects of drugs and reducing the side effects of therapeutics in the treatment of various disease conditions. Various types of DDSs have been extensively investigated as potential drug carriers for the treatment of many diseases [49,50,51,52,53,54,55,56,57,58,59,60]. For example, Wang et al. successfully constructed a simple and novel gas generator with a high drug loading level that markedly facilitated doxorubicin release and O_2_ diffusion for amplifying PDT/PTT/chemotherapy combination therapy [49]. Lei et al. constructed a multifunctional mesoporous silica nanoparticle-based drug delivery platform to load indocyanine green and doxorubicin for NIR-triggered drug release and chemo/photothermal therapy [51].

Although the applications of nanomaterials in PTT, PDT, and DDS have been great successes, improving the targeting and specificity of these therapeutic methods is still a big challenge.

## 3. Aptamer-Bound Nanomaterials Used in Different Cancer Therapies

### 3.1. Breast Cancer Therapy

The incidence of breast cancer in female cancers worldwide is 24.2%, of which 52.9% occur in developing countries [61,62]. Developing an optimized treatment method is therefore of great importance. Conventional treatments include surgery, chemotherapy, and radiotherapy. Chemotherapy and radiotherapy treatments have serious side effects. For example, during chemotherapy, the patients’ white blood cells may be reduced, and there may be problems with their blood clotting function. It will also affect the normal function of the liver. Radiotherapy is a local treatment method that uses radiation to treat tumors. This process also risks damaging normal tissues and insufficiently killing off cancer cells. Moreover, radiation therapy may also cause wound complications [63,64,65]. Therefore, the novel treatment involving binding of nanomaterials with aptamers will contribute to the development of breast cancer therapy [66,67].

#### 3.1.1. Photothermal Therapy

Molybdenum disulfide (MoS_2_) is a typical transition metal disulfide, which is a class of two-dimensional nanomaterials, that has many biomedical applications, owing to its simple preparation, good stability, large surface area, excellent water dispersibility, and biocompatibility [68,69,70]. MoS_2_ has especially high NIR absorbance. Because of this, it has high photothermal conversion efficiency. Based on this, many researchers have applied it in photothermal therapy [71,72]. However, its ability to recognize specific tumor cells needs to be improved. For example, Pang et al. developed a new method [73] which used MoS_2_ as the photothermal agent. Firstly, by mixing MoS_2_ and bovine serum albumin (BSA), researchers obtained MoS_2_-BSA nanosheets. Next, EDC and NHS were used to activate the free carboxyl group on the MoS_2_-BSA surface, and subsequently aptamers were modified to obtain composite MoS_2_-BSA-Apt nanosheets, which were stable and biocompatible. Owing to the good affinity between aptamers and receptors on the cell surface [74,75], the composition would distinguish MCF-7 human breast cancer cells from other cells and then enter the cells through endocytosis. Under the irradiation from an 808 nm laser, the heat generated kills cancer cells, which is on the basis of MoS_2_ nanosheets. After MoS_2_-BSA-Apt was cultured with MCF-7 human breast cancer cells and MCF-10A human breast cancer cells, the fluorescence-inverted microscope results showed that MCF-7 human breast cancer cells had uniformly distributed green fluorescence signals, while MCF-10A human breast cancer cells had almost no fluorescence signal. The results showed that under the same laser irradiation time, MoS_2_-BSA-Apt exhibited a better cell-killing effect than MoS_2_-BSA, indicating that MoS_2_-BSA-Apt could target MCF-7 human breast cancer cells. In addition, MoS_2_ nanosheets have magnetism, fluorescence, and other properties. These properties can be used to develop a combined accurate diagnosis and treatment platform that integrates photothermal therapy with imaging diagnosis.

**Figure 2:**
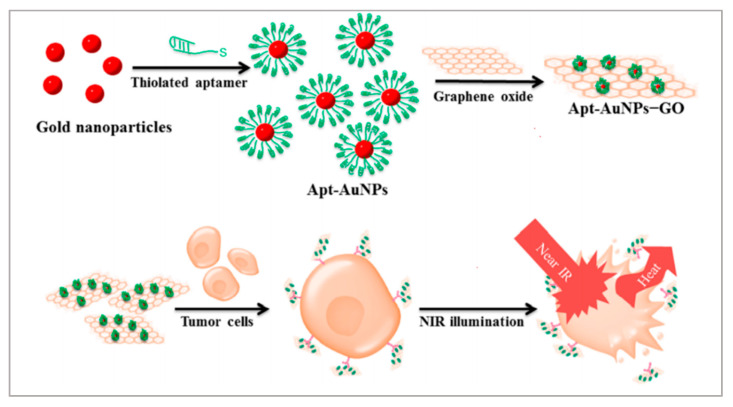
Schematic representation of the preparation of Apt-AuNP-GO and its application with NIR laser irradiation for photothermal therapy of cancer cells. Reprinted with permission from [78]. Copyright © 2015 American Chemical Society.

Graphene oxides (GOs) and gold nanoparticles (AuNPs) have been widely employed in cancer therapies because of their excellent photothermal conversion efficiencies and biocompatibilities [76,77]. GOs are excellent nanomaterials as drug carriers due to their high loading capacity. Therefore, creating a nanomatrix by combining GOs with AuNPs in a single system may enhance the photothermal effects on tumors. Considering the high loading capacities of GOs, Yang et al. anchored AuNPs on GOs to enhance the photothermal effect [78]. As shown in Figure 2, to improve the targeting capability of this nanomatrix, thiolated MUC1 aptamers were immobilized on the surface of AuNPs via strong Au−S bond, which can specifically recognize breast cancer cells. Next, the Apt–AuNPs were absorbed onto GOs. Then, the generated heat kills cancer cells with irradiation of the laser. To evaluate the targeting of Apt-AuNPs-GOs, researchers used MCF-7 cells (MUC1-positive cell lines) and EA.hy926 cells (MUC1-negative cell lines). RB molecules were loaded onto Apt-AuNPs-GOs to form fluorescent RB-Apt-AuNPs-GOs. The results indicated that EA.hy926 cells showed very weak fluorescence while MCF-7 cells showed strong red fluorescence. Owing to the excellent loading capacities of GOs, this strategy could be extended to the construction of heat shock protein inhibitor-loaded Apt-AuNPs-GOs to strengthen the effect of photothermal therapy.

**Figure 3:**
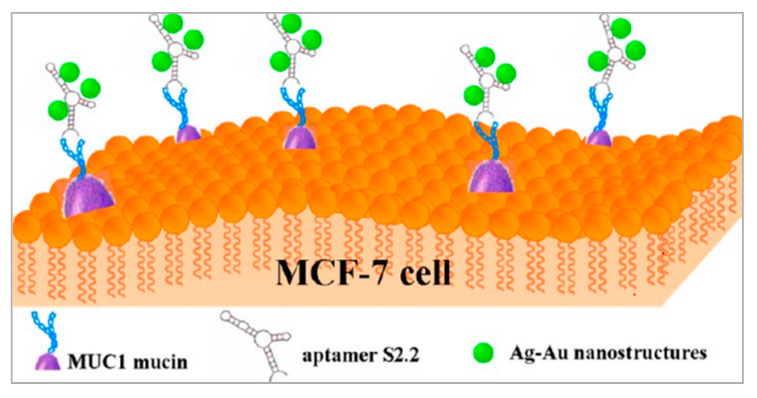
Schematic representation of the preparation of Apt-Ag-Au nanostructure and its application. Reprinted with permission from [79]. Copyright © 2012 American Chemical Society.

As shown in Figure 3, Wu et al. also proposed a method with metal nanomaterials [79]. Ag-Au nanostructures have high photothermal conversion efficiencies and are applied in PTT. By modifying the S2.2 aptamer on the surface of Ag-Au nanostructures, the Apt-Ag-Au nanostructures could interact with breast cancer cells on whose membrane MUC1 proteins are overexpressed and realized photothermal therapy. Besides, Ag-Au nanostructures are attractive surface-enhanced Raman scattering (SERS) substrates because of the synergism of these metals, the tunability of the plasmon resonance, and the superior SERS activity. The synthesized Apt-Ag-Au nanostructures will contribute to developing a protocol to specifically recognize and sensitively detect the cancer cells and facilitate the synergistic treatment of diagnosis and photothermal therapy.

#### 3.1.2. Photodynamic Therapy

Manganese dioxide (MnO_2_) has been applied more and more in biomedical areas due to its excellent loading capacities and convenient surface functionalization [80,81]. Owing to its quenching property, MnO_2_ has been used as a carrier of photosensitizer to construct novel activatable PDT systems. MnO_2_ is a unique type of tumor microenvironment-responsive nanomaterial that can react with GSH, and thus overcome the problems of PDT treatment [82,83,84]. Liu et al. proposed a new strategy [85] that used photosensitizer HMME with mesoporous MnO_2_ (mMnO_2_) functioning as the carrier of HMME. The photosensitizers were in the quenching state when loaded on the surface of mMnO_2_ nanoparticles and sealed by the aptamers on the particle surface. The aptamers were able to selectively recognize the specific membrane protein MUC1 on the tumor cell, and when this happened the photosensitizers were released. When it interacted with normal cells lacking MUC1, the HMME were not released and the PDT did not work. On the contrary, in the presence of MUC1-overexpressed breast cancer cells, the aptamer bound with MUC1 protein, and HMME was released [86,87]. Then ROS was produced under laser irradiation, which killed cancer cells. To examine the tumor-targeting release of HMME, researchers used MCF-7 cells and Hs578bst cells. The confocal laser scanning microscopy (CLSM) results showed that the fluorescence of HMME in MCF-7 cells was very bright, while it was very low in Hs578bst cells. Furthermore, after irradiation of the laser, the ROS level in MCF-7 (56.4%) was much higher than that in Hs578Bst (1.24%), which confirmed the HMME imaging results. Compared with the conventional PDT method, this constructed system provides a simple but effective approach for the selective killing of tumor cells, with infinitesimal toxicity to normal cells, and paves a new way for utilizing PDT in precise cancer treatment.

**Figure 4:**
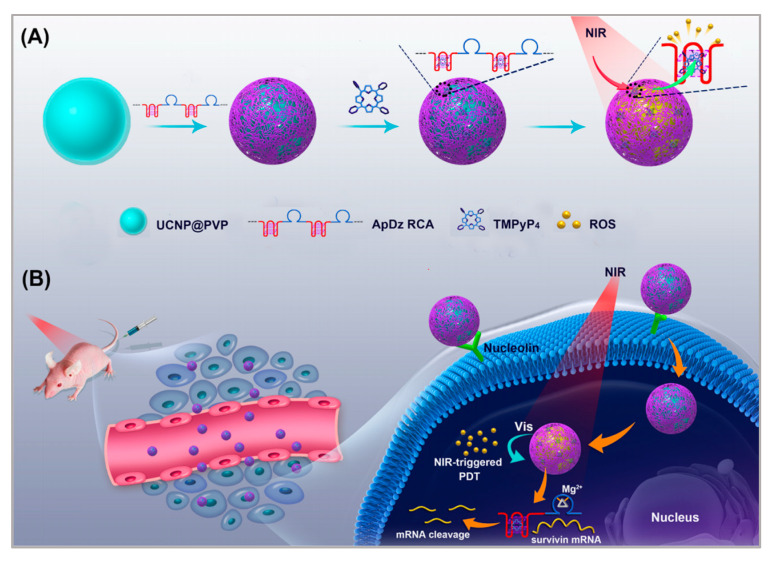
Illustration of: (**A**) the synthesis of the multifunctional DNA polymer-assisted upconversion therapeutic nanoplatform; and (**B**) the targeted photodynamic nanoplatform for highly efficient photodynamic therapy. Reprinted with permission from [88]. Copyright © 2020 American Chemical Society.

Upconversion nanoparticles (UCNPs) are often used as photosensitizer energy donors and delivery vectors in PDT therapy. Moreover, UCNPs functionalized with recognition moieties can also be conferred with the cell-targeting ability for the specific delivery of photosensitizer to enhance the efficiency of PDT. Jin et al. developed a novel method [88]. As shown in Figure 4, a long, single-stranded DNA (ssDNA) with an AS1411 aptamer and a DNAzyme was prepared using rolling circle amplification (RCA). UCNPs functioned as the carrier on which to load the ssDNA. The multivalence of the ssDNA endowed the upconversion nanoplatform with high recognition and drug loading capacity and DNAzyme inhibited the expression of survivin by gene interfering tools. In this nanosystem, AS1411 aptamer was not only used to load the photosensitizer TMPyP_4_, but it also functioned as the targeting agent to recognize the nucleolin that was overexpressed on breast cancer cells. PDT was triggered by NIR irradiation and generated ROS to kill the cancer cells. To evaluate the targeting of this nanosystem, the uptake of UCNP-ApDz-TMPyP_4_ in MCF-7 cells (the target cancer cell) and BRL 3A cells (the control cell) was detected by flow cytometry and CLSM. The CLSM results showed that the fluorescence intensity in the MCF-7 cells was significantly stronger than that of BRL 3A cells, which were consistent with the flow cytometry studies. To evaluate the cytotoxicity of MCF-7 cells, MTT assays, LIVE/DEAD viability/cytotoxicity assay were performed. The results showed that UCNP@PVP did not show any cytotoxicity, while in the UCNP-ApDz-TMPyP_4_ groups the cell survival rate was 36.3%. Emerging evidence has indicated that PDT is always adversely attenuated by the development of cancer cells resistance. However, this multifunctional upconversion nanoplatform, collaborating with PDT and DNAzyme-based gene therapy when used on tumor tissues, exhibits excellent antitumor response in vivo and in vitro and might act as an admirable alternative strategy for treating cancer.

#### 3.1.3. Drug Delivery System

Similar to the application of mMnO_2_ by Liu [85], Si et al. proposed another strategy [89]. With the help of mesoporous silica nanoparticles (MSNs), they loaded DNA sensor-capped doxorubicin (DOX). DNA sensors on the targeted nanoparticles could trigger DOX release through a conformational switch induced by MUC-1 protein. They modified the aptamer on the surface of MSNs, which can recognize MUC-1 protein [90]. When the composition was endocytosed into MCF-7 cells, in which MCU-1 was overexpressed, DOX would be released and would kill cancer cells. This caused a significant difference in cell viability between breast cancer MCF-7 and normal breast Hs578bst cells (24.8% and 86.0%). The selectivity and efficiency of treatment were improved greatly. Although the MUC-1 adaptor has been used for drug delivery, the adaptor only served as a targeted ligand and could not accomplish the controlled release of drugs. In this nanosystem, DOX release could be specifically “turned on” in tumor cells according to the MUC1-induced conformational change. This nanosystem provides a new idea for the drug delivery system.

**Figure 5:**
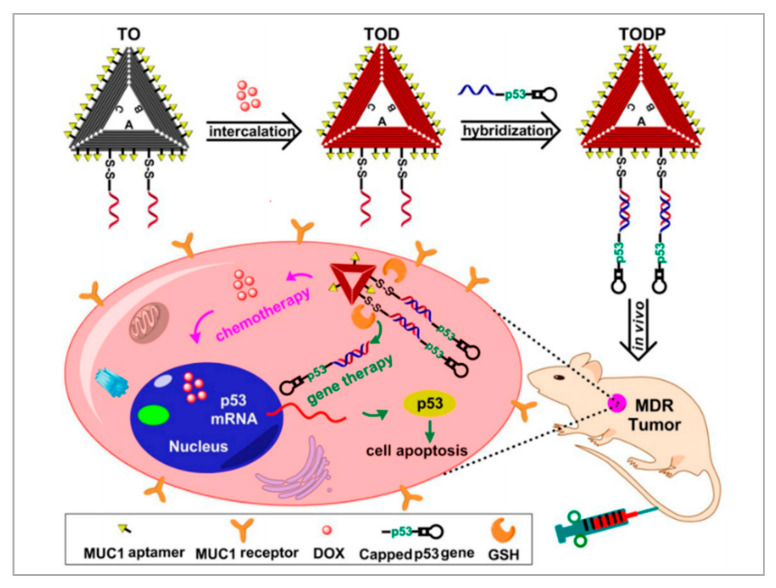
Schematic illustration showing the DNA nanostructure-based combination of gene therapy and chemotherapy. Reprinted with permission from [91]. Copyright © 2018 American Chemical Society.

Gene therapy is a promising therapeutic strategy to combat many serious gene-related diseases. Liu et al. developed a novel drug delivery system to combine gene therapy with chemotherapy [91]. During long-term chemotherapy, tumors show drug resistance. Researchers have found that the p53 gene can enhance the sensitivities of drug-resistant tumors to chemotherapeutics. It is well known that DNA nanostructure can be designed to assemble a variety of functional components. As shown in Figure 5, a biocompatible triangle DNA origami was chosen to efficiently load DOX (TOD) and p53 genes (TODP), and then the MUC1 aptamer was modified on the surface of the DNA nanostructure to improve targeted delivery and controlled release. To evaluate the targeting of TODP, delivery vectors with aptamers and without aptamers were used. The biodistribution of these delivery vectors was studied in an animal imaging system utilizing the fluorescent signal of Cy5.5-labeled DNA origami. The results showed that those with aptamers located mainly in the tumor tissue had substantially higher intensity than those without aptamers. Furthermore, with the application of DNA nanostructure, additional functional groups such as RNA-based drugs, gene editing systems, and imaging diagnosis components may also be introduced into this codelivery system for synergistic theranostics. We think this is a promising platform for the development of a new generation of therapeutics for the treatment of cancers.

#### 3.1.4. Photothermal Therapy/Photodynamic Therapy/Chemotherapy

**Figure 6:**
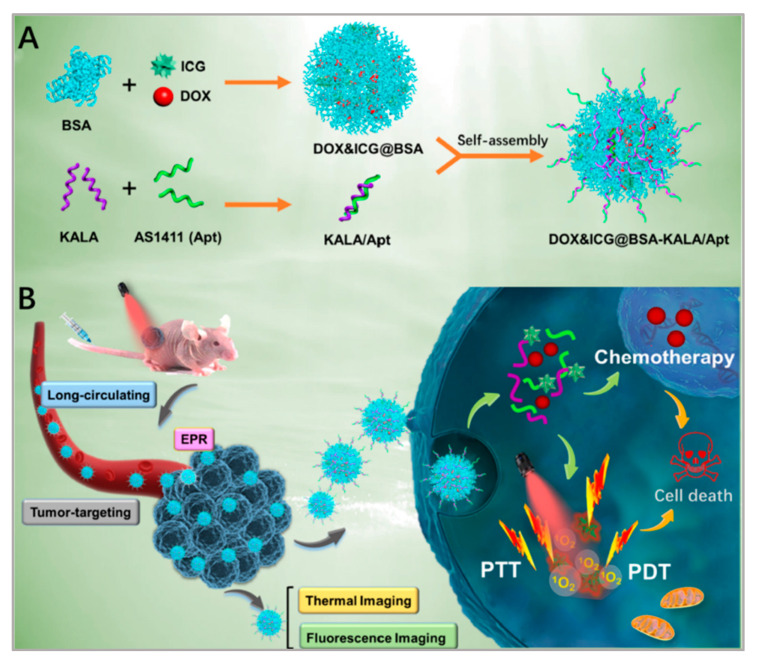
Schematic illustration of: (**A**) the preparation of DOX/ICG/BSA/KALA/Apt nanoparticles; and (**B**) the theranostic process based on DOX/ICG/BSA/KALA/Apt nanoparticles. Reprinted with permission from [92]. Copyright © 2019 American Chemical Society.

Among various combined treatments, combined chemotherapy/phototherapy is a practicable and promising strategy for cancer treatments due to its favorable synergistic effects and clinical realizability. Xu et al. developed a strategy to combine PTT, PDT, and chemotherapy [92]. As shown in Figure 6, they assembled DOX, indocyanine green (ICG), and bovine serum albumin (BSA) molecules to form nanosized DOX/ICG/BSA nanoparticles. To improve the targeting of the nanoparticles, AS1411 aptamers and a cell-penetrating peptide (KALA) were modified on the surface of the DOX/ICG/BSA nanoparticles through electrostatic interaction. Finally, under the irradiation of the laser, phototherapy was applied and DOX was released to realize chemotherapy. Researchers chose nucleolin overexpressed MCF-7 cells to study the targeting of DOX/ICG/BSA/KALA/Apt. After 4 h of incubation, compared with DOX/ICG/BSA, DOX/ICG/BSA/KALA/Apt showed higher intracellular concentrations of both DOX and ICG. However, in no nucleolin overexpressed 293T cells, the cellular uptakes of DOX/ICG/BSA and DOX/ICG/BSA/KALA/Apt were nearly the same. Besides, the cytotoxicity of DOX/ICG/BSA/KALA/Apt was stronger than DOX/ICG/BSA in MCF-7 cells. Studies showed improved antitumor efficiency of DOX/ICG/BSA/KALA/Apt nanoparticles and demonstrated that the functional theranostic system had great promise in tumor treatment. Although integrated therapeutic systems have developed a lot, the toxicity, biodegradability, and cumbersome assembly processes are still big problems. In future study, researchers should focus on the facile and biocompatible assembly of multifunctional nanosystems.

### 3.2. Lung Cancer Therapy

Lung cancer is one of the malignant tumors with the fastest increase in morbidity and mortality, and therefore lung cancer is the greatest threat to human health and life [3]. Many countries have reported that the incidence and mortality of lung cancer have increased significantly. Among all malignant tumors, both the incidence and mortality of lung cancer are highest in men [93,94]. Therefore, the development of an efficient treatment strategy is urgently needed. Chemotherapy is the dominant treatment method among conventional treatments. Though chemotherapy can work in the initial stage, cancer cells will develop drug resistances, while its side effects are unavoidable, causing damage to normal cells [95,96]. Hence, developing an efficient, specific, and less toxic cancer cell treatment platform is the problem that needs to be addressed.

#### 3.2.1. Drug Delivery System

Researchers have made great efforts to develop a range of drug delivery systems [97,98,99]. It is well known that magnetic particles have many biomedical applications [100,101,102,103,104], such as detection [105,106], drug delivery [107], etc. Magnetic materials, especially Fe_3_O_4_, have been considered as effective drug delivery systems due to their hollow structures. Moreover, Fe_3_O_4_ is also used as a PTT agent. Zhao et al. used Fe_3_O_4_ nanoparticles (Fe_3_O_4_ NPs) as the carrier to load DOX to realize combined chemotherapy/PTT. A carbon layer was applied to cover the magnetite to improve its stability and photothermal conversion efficiency [108]. To improve the targeting of Fe_3_O_4_/carbon/DOX nanoparticles (Fe_3_O_4_/C/DOX NPs), the aptamers (sgc8) were modified on the surface of Fe_3_O_4_/C/DOX NPs. Furthermore, magnetite is sensitive to acidic conditions. Therefore, under laser irradiation and with a pH of about 5.5, the released DOX and generated heat will kill cancer cells. The CLSM results showed that the fluorescence of A549 cells treated with Apt/Fe_3_O_4_/C/DOX NPs was significantly enhanced compared with that of the Fe_3_O_4_/C/DOX NPs alone, which suggests that the sgc8 aptamer facilitated the uptake of NPs. The results in vitro and in vivo demonstrated that the targeted chemo–photothermal combination therapy led to the complete eradication of tumors. As Fe_3_O_4_ is frequently used as a contrast agent for T_2_-weighted magnetic resonance (MR) imaging for diagnostic and therapeutic applications, it is promising to combine imaging diagnosis and treatment in this nanosystem. Furthermore, considering the large effective surface area of Fe_3_O_4_, more functional groups can be used to develop multidrug loading.

#### 3.2.2. Photodynamic Therapy

**Figure 7:**
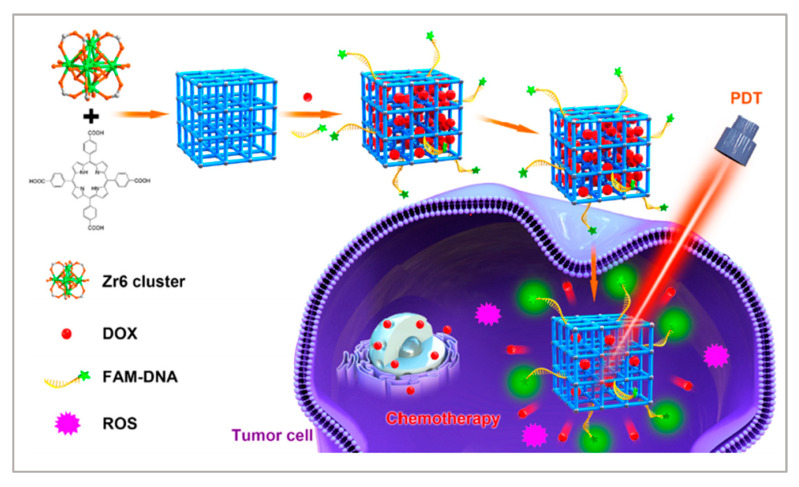
Schematic representation of DNA-functionalized NMOFs for targeted drug delivery and PDT. Reprinted with permission from [109]. Copyright © 2019 American Chemical Society.

PDT is also widely applied in lung cancer therapy. Nano metal-organic frameworks (NMOFs) have received attention for their wide applicability in drug loading and delivery. Owing to their structural advantages, NMOFs are capable of controlling drug interactions with biological systems, and the drug loading efficiency is affected by the morphology and size of NMOFs. The coordination bands of NMOFs are also weak, which contribute to their biodegradability and expand their clinical applications. Zhang et al. developed a novel method to realize the combination of chemotherapy with PDT [109]. Figure 7 shows a representation of how they first synthesized NMOFs. To achieve an excellent drug loading efficiency of NMOFs, they used NMOFs to load DOX, which is prepared for chemotherapy. Furthermore, they also modified the aptamers of A549 cells on the surface of NMOFs, which can distinguish between A549 cells and normal cells. After the irradiation of the laser, DOX and ROS will kill cancer cells when released. Alongside simultaneous treatment with chemotherapy and PDT, the viability was 30% for A549 cells, whereas it was 45% for MCF-7 cells, indicating PDT and chemotherapy’s targeting effects for A549 cells of this nanosystem. Moreover, the CLSM results also showed that the fluorescence of DOX was obvious for the A549 cells while weak for the MCF-7 cells. The facile aptamer functionalization of NMOFs offers an opportunity to develop target-directed therapeutic nanosystems. Notably, the aptamer was modified with fluorescein at the terminus to trace A549 cells by targeting nanosystems towards the cells. Based on this, researchers are encouraged to facilitate the integration of diagnosis and treatment nanosystems.

**Figure 8:**
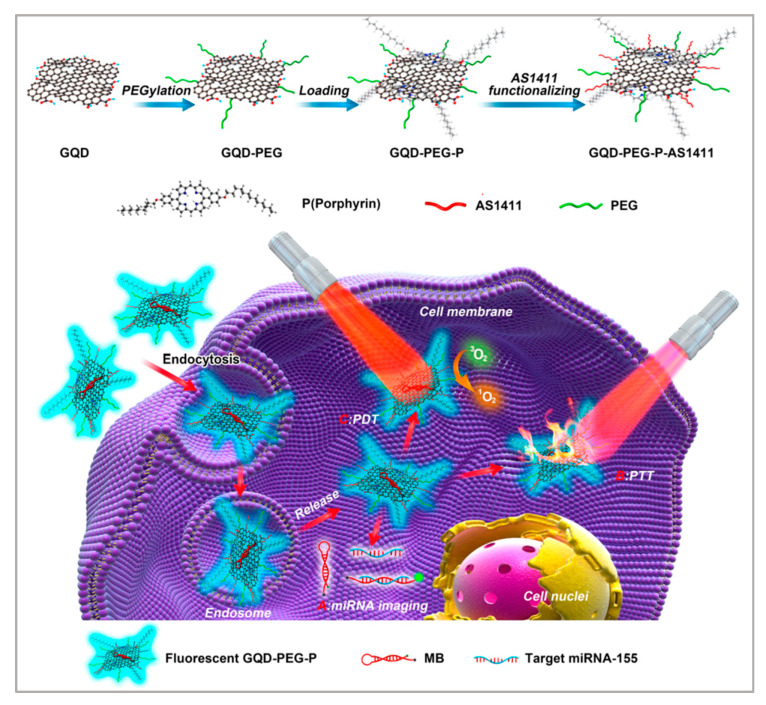
Synthesis of GQD-PEG-P for the combined photothermal/photodynamic therapy. Reprinted with permission from [110]. Copyright © 2016 American Chemical Society.

Owing to their ultra-high surface areas, graphene quantum dots (GQDS) have emerged as effective cargo nanovectors for drug loading. Moreover, GQDS are widely used in phototherapy owing to the high NIR absorption. Meanwhile, GQDs are relatively common as bioimaging and fluorescent labels because of their biocompatibility. Based on the enormous unique physicochemical properties of GQDS, Cao et al. developed another method [110]. As shown in Figure 8, they used PEGylated GQDS to load porphyrin-derivative photosensitizers, and then AS1411 aptamers were modified on the surface of GQDs so that the nanosystem could selectively target A549 cells. In this nanosystem, GQDs functioned as the drug carrier and photothermal agent. Under the irradiation of the laser, PTT and PDT occurred. The toxic ROS and the heat generated killed the cancer cells. Researchers used HDF cells and A549 cells to verify the targeting of this nanosystem. After irradiation of the laser, almost all the A549 cells were apoptotic, while HDF cells showed no apoptosis. Furthermore, this nanosystem could realize intracellular miRNA biomarker detection and fluorescence-guided PTT/PDT synergetic therapy, which holds great potential in developing combined diagnostics with therapeutics. Notably, chemotherapy can be integrated into this nanosystem with the high drug loading efficiency of GQDs to enhance the treatment effect.

### 3.3. Liver Cancer Therapy

Liver cancer is one of the most common malignant tumors worldwide. It ranks fifth in incidence rate and second in fatality rate in China. About half of the newly diagnosed cases in the world come from China. According to relevant predictions of the World Health Organization, the number of deaths due to liver cancer in 2030 will reach one million. Globally, liver cancer has the third highest fatality rate, second only to lung cancer and gastric cancer [3]. Liver cancer is difficult to detect at the early stage and develops rapidly, however, various devices developed recently may be modified and applied to detect it [111,112,113]. Conventional treatments of liver cancer include surgical resection, liver transplantation, radiotherapy, and chemotherapy, but these treatments suffer from side effects of varying degrees.

#### Photodynamic Therapy

**Figure 9:**
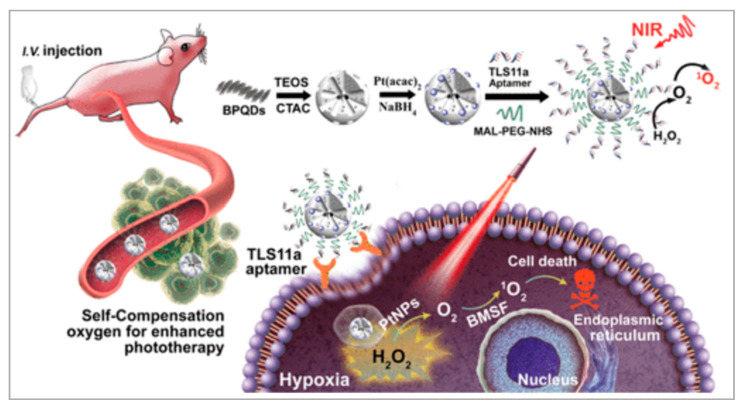
Schematic illustration of the self-regulation of precise cancer phototherapy. Reprinted with permission from [114]. Copyright © 2019 American Chemical Society.

In recent decades, black phosphorus quantum dots (BPQDs) have been widely applied in biomedical areas, owing to their excellent photocatalysis activities in PDT and broad photo-absorption in PTT. Lan et al. proposed a PDT method to treat liver cancer [114]. As shown in Figure 9, they firstly synthesized a BPQDs-hybridized mesoporous silica framework (BMSF) and Pt nanoparticles (PtNPs). After TLS11a aptamer was decorated on the surface of BMSF-Pt, the Apt-BMSF-Pt could actively target liver cancer cells. Following irradiation, the Apt-BMSF-Pt would generate ROS to kill cancer cells. After HepG2 cells were incubated with Apt-BMSF-Pt and BMSF-Pt, several apoptosis cells could be observed in BMSF-Pt-treated HepG2 cells in the presence of laser irradiation. In contrast, a large number of dead cells were observed in Apt-BMSF-Pt-treated HepG2 cells with laser irradiation, attributing to the efficient uptake by aptamer-mediated endocytosis. The in vivo studies also indicated that Apt-BMSF-Pt had a better tumor growth inhibition effect than BMSF-Pt. Significantly, the BMSF-Pt could self-supply oxygen under H_2_O_2_ conditions to enhance PDT. Because the tumor microenvironment is hypoxic, this nanoplatform can be developed into a targeting nanocatalyst for the self-regulation of precise cancer phototherapy.

### 3.4. Cervical Cancer Therapy

Cervical cancer is one of the most common female malignancies. According to statistics, there are about 570,000 new cervical cancer patients worldwide each year, and about 310,000 people die from the disease. Cancer-related mortality is high among all gynecological malignancies. Cervical cancer is ranked first among all female malignant tumors, and fourth among all female malignant tumor-related death rates [3]. Early cervical cancer is generally treated with surgery, but the overall prognosis of patients with recurrence and advanced cervical cancer is still not optimistic. Therefore, developing an efficient treatment method is a big challenge.

#### 3.4.1. Drug Delivery System

Compared with conventional passive targeting in the tumor vasculature, an active targeting strategy via the specific binding of ligands to tumor markers greatly improves intracellular accumulation. Zhang et al. proposed a nanosystem to combine tumor imaging and drug delivery [115]. Persistent luminescence nanoparticles (PLNPs) have aroused widespread interest in fluorescence bioimaging because of their unique optical properties. They used PLNPs as the core, which contributed to tumor imaging. Polyamide-amine (PAMAM) was then modified on the surface of PLNPs to provide many groups for further AS1411 aptamer functionalization, while DOX was loaded onto the nanoplatform via an acid-sensitive hydrazone bond (PLNPs-PAMAM-AS1411/DOX). To examine the targeted cellular uptake of PLNPs-PAMAM-AS1411/DOX, HeLa cells (overexpressed nucleolin) and 3T3 cells (low nucleolin expression) were selected. CLSM imaging and flow cytometry analysis showed a much stronger luminescence signal compared to 3T3 cells after incubation with PLNPs-PAMAM-AS1411/DOX. The in vivo studies also indicated that PLNPs-PAMAM-AS1411/DOX had a better tumor growth inhibition effect than PLNPs-PAMAM-DOX. The intracellular-controlled release of DOX via a pH-sensitive hydrazone killed tumor cells effectively. Owing to the AS1411 aptamer, the nanoplatform had excellent specificity for tumor cells, whose membrane nucleolin were overexpressed [116,117]. This nanoplatform has the potential for bioimaging and the treatment of tumors with high specificity and efficiency.

#### 3.4.2. Photodynamic Therapy

**Figure 10:**
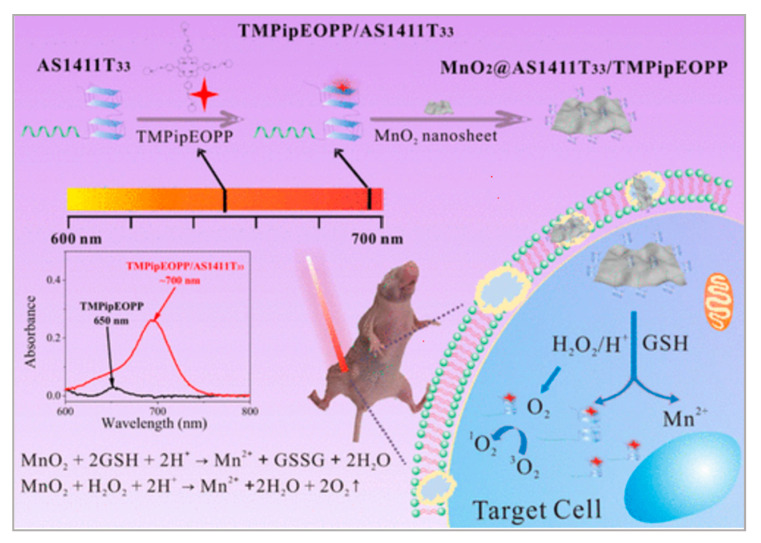
Preparation of G-quadruplex/porphyrin composite photosensitizer and its application in PDT treatment of solid tumors after assembly with MnO2 nanosheet. Reprinted with permission from [118]. Copyright © 2019 American Chemical Society.

Apart from an improved drug delivery system, PDT was also applied in cervical cancer therapy. Cheng et al. reported a novel strategy to develop a kind of hydrophilic photosensitizer with good tumor specificity and high ROS generation efficiency under the stimulation of NIR light [118]. In this work, they mixed DNA G-quadruplexes with a hydrophilic porphyrin (TMPipEOPP)^4+^·4I^−^. MnO_2_ was used to consume GSH in order to reduce ROS consumption and generate O_2_ to enhance PDT. As shown in Figure 10, when the G-quadruplex/porphyrin complexes were further assembled with AS1411 aptamer and MnO_2_ nanosheets, CLSM results showed that HeLa cells incubated with MnO_2_/AS1411/TMPipEOPP showed brighter red fluorescence than TMPipEOPP alone, demonstrating that MnO_2_/AS1411/TMPipEOPP could be efficiently internalized into cancer cells when combined with AS1411 aptamers. The in vivo experiments showed better tumor growth inhibition in MnO_2_/AS1411/TMPipEOPP + NIR groups when compared with TMPipEOPP + NIR groups. As an essential part of PDT, this work proposed a facile way to improve the penetration depth and PDT efficacy of photosensitizers. Based on this, researchers can try to prepare more kinds of photosensitizers to develop PDT.

**Figure 11:**
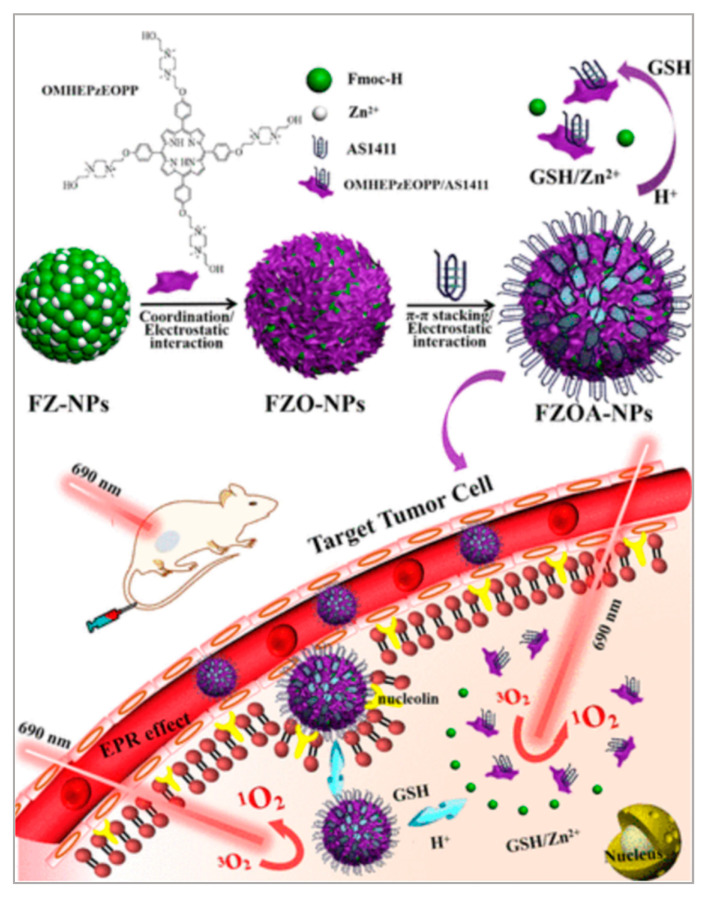
Preparation of FZOA-NPs and their application in tumor PDT treatment. Reprinted with permission from [119]. Copyright © 2020 American Chemical Society.

Presently, due to their attractive biological optical properties, porphyrin compounds have been widely used as photosensitizers in PDT. To improve their limited tumor accumulation, many delivery systems have been developed, but biocompatibility is still a big problem. As shown in Figure 11, Chu et al. proposed a strategy [119]. They first synthesized Fmoc-H/Zn^2+^ nanoparticles (FZ-NPs) to load porphyrin/Gquadruplex composite photosensitizers (FZO-NPs), and then the AS1411 aptamer was modified on the surface of FZO-NPs (FZOA-NPs). With the NIR light irradiation, ROS would be produced to kill cancer cells. The nanoassembly which they prepared showed a great potential in tumor treatment, and this has been demonstrated in both in vitro and in vivo experiments. Notably, the preparation process only involved amino acid, nucleic acid, Zn^2+^, and biocompatible porphyrin, obviously reducing the potential for toxic effects to occur during the synthesis process, which was beneficial to the further application. Overall, this study provided a simple and green way to prepare highly biocompatible and highly efficient NIR nanocomposite photosensitizers for clinical tumor PDT treatment.

### 3.5. Gastric Cancer Therapy

Gastric cancer is one of the most common malignant tumors, and is a leading cause of cancer-related death worldwide [3,120]. Although researchers have made great effort to develop cancer therapy methods, the treatment efficiency is still not satisfactory. Currently, the dominant method is to use chemical drugs, but the specificity and reverse side effects are problems that remain to be solved [121,122].

Gold nanoparticles (AuNPs) are used more and more widely for their highly efficient internalization and good biocompatibility [123,124,125,126]. Moreover, AuNPs are easily modifiable with targeting ligands, and have their own high photothermal conversion efficiency. Zhang et al. developed a new method to treat gastric cancer [127]. Researchers modified the AS1411 aptamer on the surface of AuNPs. AS1411 aptamer can strongly bind to nucleolin, which is a new molecular target for gastric cancer treatment [128,129,130]. The nanosystem, therefore, showed tumor-specific targeting. Furthermore, with the modification of hairpin DNA, DOX was loaded by intercalation. Under the irradiation of the laser, the generated heat and drug release exhibited excellent cancer therapy effects. AGS cells (high nucleolin expressing) and L929 cells (low nucleolin expressing) were used to verify the targeting of this nanosystem. CLSM results showed that the red fluorescence was almost parallel in both AGS and L929 cells incubated with free DOX. When cells were treated with AS1411-based nanoparticles, stronger red fluorescent intensity was observed in the AGS cells, but less in the L929 cells. This multifunctional nanosystem may be promising for gastric cancer cells treated in the future.

### 3.6. Colorectal Cancer Therapy

Colorectal cancer is the third most frequently diagnosed cancer and its fatality rate is very high, especially in many developing countries [3]. In recent years, researchers found that the incidence age of colorectal cancer is getting younger and younger. Moreover physical inactivity and smoking are the most common factors in the occurrence of colorectal cancer [131]. Therefore, it is urgent to look for an effective treatment method.

With the application of AuNPs, Go et al. developed one strategy [132] where cellular prion protein PrP^C^, which is demonstrated to be overexpressed in colorectal cancer, functioned as the site of action [133,134,135]. They modified PrP^C^ aptamer on the surface of gold nanoparticles, followed by hybridization of its complementary DNA for DOX loading. The flow cytometry results revealed that the uptake was significantly increased in PrP^C^-positive cells compared to PrP^C^-negative cells. The aptamer will bind to PrP^C^ specifically and thus improve the drug delivery efficiency. Under laser irradiation, the heat generated and the DOX released will synergistically kill cancer cells. This nanosystem can serve as an effective therapeutic agent for colorectal cancer treatment.

### 3.7. Prostate Cancer Therapy

The incidence of prostate cancer ranks fourth among malignant tumors and second among male malignancies, second only to lung cancer. The mortality rate of prostate cancer is in the top ten among malignant tumors and the fifth among male malignant tumors [3]. The pathogenesis of prostate cancer is not yet clear, and the incidence is increasing year by year, and the growth rate ranks second among all tumors.

**Figure 12:**
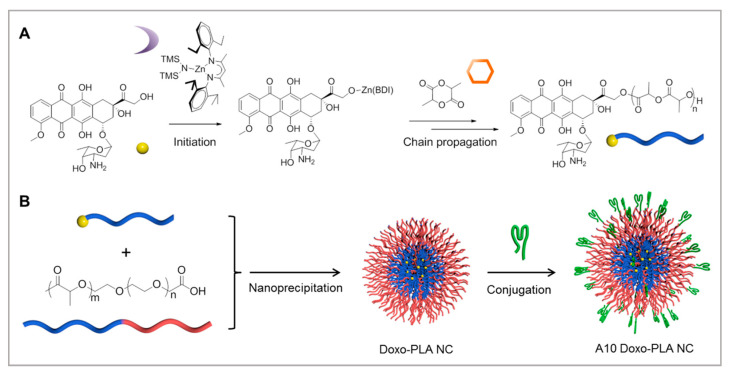
Preparation of A10 aptamer-functionalized doxorubicin-polylactide nanoconjugates (A10 Doxo-PLA NCs): (**A**) synthesis of Doxo-PLA polymer conjugate; (**B**) schematic illustration of formulating A10 Doxo-PLA NCs. Reprinted with permission from [136]. Copyright © 2015 American Chemical Society.

Prostate-specific membrane antigen, a protein that overexpresses in prostate cancers, is an ideal target for the treatment of prostate cancer. Tang et al. proposed a new method for treatment [136]. As shown in Figure 12, they firstly developed doxorubicin-polylactide nanoconjugates (DOX-PLA NCs) for drug delivery, which would reduce the dose-limiting toxicities associated with free DOX. A10 aptamer, which can target prostate cancer, was immobilized on the surface of DOX-PLA NCs to improve the targeting. Both in vitro and in vivo results showed that controlled drug release would kill cancer cells. The improved efficacy and reduced systemic toxicity of the targeting A10-DOX-PLA NCs demonstrated their strong potential for future clinical translation.

## 4. Commonly Used Nanomaterials Modified with Aptamers in Cancer Therapy

### 4.1. Gold Nanorods

Owing to their high photothermal conversion efficiency, gold nanorods (AuNRs) have been studied extensively [137,138,139]. Compared with nanospheres or nanoshells, AuNRs sustain strong absorption of NIR light, which is less easily absorbed by normal tissues and, therefore, provides deep-tissue penetration with high spatial precision, thus causing minimal damage to healthy tissue. As good candidates for PTT, the precise targeting of AuNRs is, indeed, of crucial importance to their effectiveness. The introduction of aptamers will greatly increase the photothermal killing effect.

Cheng et al. discovered an aptamer (KW16-13) that can specifically recognize MCF10CA1h breast cancer cells [140]. To replace the hexadecyltrimethylammonium bromide (CTAB) with PEG, mPEG-SH solution was added to the prepared AuNRs. Next, a thiolated aptamer was modified on the surface of PEG-AuNR, so that the Apt-AuNR could target the tumor cell. Then, with the irradiation of the laser, the heat generated could kill the cancer cells. Compared with AuNRs, Apt-AuNRs exhibited a better killing effect. AuNRs conjugated to KW16-13 aptamers were readily internalized by the MCF10CA1h tumor cells with minimal uptake by MCF10A normal cells. Upon NIR light irradiation, tumor cell death of >96% could be effected, compared to <1% in the normal cells. The high tumor-cell specificity exhibited under the direction of the KW16-13 aptamer made them exciting candidates for development as novel anti-tumor therapeutics.

Zheng et al. developed a new strategy with the help of exosomes to transport gold nanorods, which possess photothermal conversion properties [141]. Exosomes are nanosized membrane vesicles endogenously secreted by cells, and they can mediate intercellular communication [142,143]. Moreover, they have excellent biocompatibility, blood circulation, and nearly have non-immunogenicity, which contributes to their quantitative use in biomedical applications [144,145]. Firstly, the exosomes were incubated with DSPE-PEG-SH and double-face-adhesive TLS11a aptamers. Thus, the exosome membranes were modified with both sulfhydryl groups and targeting aptamers. Finally, the AuNRs could be easily combined with the exosomes through the formation of Au-S bonds (Apt-Exos-AuNRs). Through the tight linkage of exosomes, AuNRs, and aptamers, it is possible to achieve both specific targeting and selective photothermal destruction of cancer cells. The fluorescence imaging revealed that the HepG2 cells were found to exhibit broader red fluorescence than the L02 cells after incubating CM-Dil-labeled Apt-Exos-AuNRs with cells, which verified the targeting capabilities of this nanosystem. Moreover, the introduction of exosomes could not only promote the internalization of AuNRs into cells, but the exosome itself could be used as a potential drug delivery vehicle for diverse clinical applications in the future.

### 4.2. PLGA Nanoparticles

PLGA nanoparticles are widely applied in biomedical areas for their good biocompatibility, and their degradation products are lactic acid and glycolic acid, which are relatively nontoxic. Based on this, the PLGA nano matrix is frequently used as a nano-drug carrier to release the drug in the tumor site in response to various stimuli. Although this strategy has achieved a great success, targeting is still a big problem. By modifying aptamers, the delivery efficiency has been improved a lot.

Duan et al. developed a nanoparticle-based drug delivery system to treat triple-negative breast cancer (TNBC) [146]. Researchers found that heparinase (HPA) was highly expressed in TNBC. Duan et al. used PEG-functionalized PLGA nanoparticles to load paclitaxel, a drug to treat breast cancer, and then HPA aptamers (S1.5) were modified on the surface of nanoparticles. The aptamers specifically targetted HPA, with paclitaxel to be released to kill cancer cells. In future research, HPA could potentially be one of the recognized molecular targets for TNBC therapy, and this targeted drug delivery aimed towards HPA may serve as a potential method to improve the efficiency of TNBC treatments.

Wang et al. put forward a new strategy [147], where PLGA nanoparticles were used to function as a carrier to load DOX. Poly (N-vinylpyrrolidone) (PVP) was then used to stabilize PLGA nanoparticles. To improve the specificity of DOX-PLGA-PVP NPs, aptamer AS1411 was modified on the surface of DOX-PLGA-PVP NPs, to enable it to target lung cancer cells [148,149]. When the aptamer reacted with a receptor on the cancer surface, APT-DOX-PLGA-PVP NPs could enter the cell by endocytosis. Afterwards, when pH was regulated, DOX was released to kill cancer cells. The results of flow cytometer-based analysis indicated the higher cellular uptake of the DOX released from the APT-DOX-PLGA PVP NPs, which was higher than the DOX alone treated cells. The in vivo results also showed that APT-DOX-PLGA-PVP NPs had a better tumor growth inhibition effect than free DOX alone, attributable to the AS1411 aptamers. Overall, this novel drug delivery system can enhance cancer therapy by targeting the nucleolin receptor endocytosis.

Zhang et al. developed another method based on PLGA nanoparticles [150]. Homoharringtonine (HHT), an effective anticancer agent, was loaded by PLGA nanoparticles. Next, epidermal growth factor receptor (EGFR) aptamer was modified on the surface of PLGA nanoparticles, which enabled it to specifically recognize EGFR in lung cancer cells. As the level of GSH increased, the PLGA nanomedicine was triggered and HHT was released to kill cancer cells. Compared with a free anticancer drug, experiments indicated that the PLGA nanomedicine has better therapeutic efficacy. Therefore, owing to the high loading efficiency of PLGA nanoparticles, this nanoplatform has the possibility to load a combination of drugs and to serve as a powerful tool for therapies for various types of malignant tumors.

We list several commonly used nanomaterials in cancer therapies (Table 1).

**Table 1:** Summary of Aptamer-Bound Nanomaterials in Cancer Therapy.

Aptamer	Nanomaterial	Application	References
MS03 aptamer	Molybdenum disulfide	Breast cancer therapy	[73]
KW16-13 aptamer	Gold nanorods	Breast cancer therapy	[140]
MUC1 aptamer	Gold nanoparticles/Graphene oxides	Breast cancer therapy	[78]
S2.2 aptamer	Ag-Au nanostructure	Breast cancer therapy	[79]
MUC1 aptamer	Mesoporous MnO_2_	Breast cancer therapy	[85]
AS1411 aptamer	Upconversion nanoparticles	Breast cancer therapy	[88]
MUC1 aptamer	Mesoporous silica nanoparticles	Breast cancer therapy	[89]
S1.5 aptamer	PLGA nanoparticles	Breast cancer therapy	[146]
MUC1 aptamer	DNA nanostructure	Breast cancer therapy	[91]
AS1411 aptamer	DOX/ICG/BSA nanoparticles	Breast cancer therapy	[92]
Sgc8 aptamer	Fe_3_O_4/_Carbon nanoparticles	Lung cancer therapy	[108]
AS1411 aptamer	PLGA nanoparticles	Lung cancer therapy	[147]
EGFR aptamer	PLGA nanoparticles	Lung cancer therapy	[150]
Aptamer of A549 cell	Nano metal-organic frameworks	Lung cancer therapy	[109]
AS1411 aptamer	Graphene quantum dots	Lung cancer therapy	[110]
TLS11a aptamer	Gold nanorods	Liver cancer therapy	[141]
TLS11a aptamer	Black quantum dots/Mesoporous silica framework/Pt nanoparticles	Liver cancer therapy	[114]
AS1411 aptamer	Persistent luminescence nanoparticle	Cervical cancer therapy	[115]
AS1411 aptamer	Manganese dioxide nanosheets	Cervical cancer therapy	[118]
AS1411 aptamer	Fmoc-H/Zn^2+^/OMHEPzEOPP nanoparticles	Cervical cancer therapy	[119]
AS1411 aptamer	Gold nanoparticles	Gastric cancer therapy	[127]
PrP^C^ aptamer	Gold nanoparticles	Colorectal cancer therapy	[132]
A10 aptamer	Polylactide nanoconjugates	Prostate cancer therapy	[136]

## 5. Conclusions

In the past few decades, researchers have made great efforts to look for new methods for cancer treatment. Extensive research on the combination of aptamers with nanomaterials has worked a lot in cancer treatment. This review has summarized several applications of aptamers in cancer treatment. The greatest contribution of aptamers in cancer treatment nanoplatforms includes their convenient modification on the surface of nanomaterials and their specific recognition of and binding to targets. Firstly, easy construction of nanoplatforms will have wide and beneficial applications in the future. Secondly, owing to the excellent specificity of aptamers, cancer treatment based on aptamer-modified nanomaterials will do less harm to normal cells and improve treatment efficiency. Finally, considering the many unique properties of nanomaterials, they can function as the medium for photothermal therapy and photodynamic therapy, and as a carrier to load drugs. As more and more properties of nanomaterials are studied, the combination of aptamers with nanomaterials will be applied in more areas that are not covered.

Although the combination of aptamers with nanomaterials has developed a lot in cancer treatment, most research still stopped at the animal level. As is known, the treatment methods must be tested in the human body within ethical approval for the clinical application of these aptamers. Therefore, researchers must further testify to their effectiveness. Furthermore, the current research scope is focused on cancer treatment, and therefore researchers should study other fields to expand the applications of aptamer-embedded nanomaterials, such as tumor imaging, cancer diagnosis, and so on. In the current research, researchers take advantage of different unique properties of nanomaterials, but there are still many properties that we do not know about, and researchers can try to develop this to find further applications for aptamer-embedded nanomaterials. Moreover, for safety, biocompatibility is one of the most important issues. Compared with conventional antibodies, the established technology of aptamers is improving and their manufacturing cost is high. Therefore, researchers should further invest in aptamer technology in the future, as more and more aptamers are selected, as we expect they will be. As the aptamer technology is developing, more aptamers will be used in biomedical applications, which will improve the accuracy of diagnosis and effectiveness of treatment in cancer.

Despite all of the challenges, the applications of aptamers in cancer therapy are moving in the direction of future treatment development. With the development of different subjects, aptamer-embedded nanomaterials will have further improvement.

## Data Availability

Data is contained within the article.
